# “Streets of Fire” revisited: contact burns

**DOI:** 10.1186/s41038-019-0169-9

**Published:** 2019-10-29

**Authors:** Areta Kowal-Vern, Marc R. Matthews, Karen N. Richey, Kathy Ruiz, Michael Peck, Arpana Jain, Kevin N. Foster

**Affiliations:** 1 Arizona Burn Center, Maricopa Medical Center, Department of Surgery, Maricopa Integrated Health System, 2601 E. Roosevelt Street, Phoenix, Arizona 85008 USA; 2 Research Department, Maricopa Integrated Health System, Phoenix, Arizona 85008 USA

**Keywords:** Pavement, Concrete, Asphalt, Contact burns, Road rash, Streets, Highways, Sand, Rocks

## Abstract

**Background:**

Pavement-street contact burns are rare. This study compared recent contact burns to those published in “Pavement temperature and burns: Streets of Fire” in 1995. The hypothesis was that there were a significantly increased number of pavement-street burns, as a result of increased ambient temperatures, and that motor vehicle crash (MVC) contact burns were less severe than pavements-street burns.

**Methods:**

This was a retrospective burn center registry study of naturally heated surface contact burns during May to September from 2016 to 2018. Statistical analyses were performed with one-way analysis of variance (ANOVA) and Maximum Likelihood chi-squared for age, percent of total burn surface area (% TBSA), treatment, hospitalization, comorbidities, hospital charges, mortality, ambient, and surface temperatures (pavement, asphalt, rocks).

**Results:**

In the 1995 study, median ambient temperatures were 106 (range 100–113) °F compared to the 108 (range 86–119) °F highest noon temperature in the current study. No ambient temperature differences were recorded on days with pavement burn admissions compared to days without these admissions. There were 225 pavement, 27 MVC, 15 road rash, and 103 other contact burns. The major injuries in the pavement group were due to being “down” (unknown reason), falls, and barefoot. Compared to the others, the pavement group was older, 56+ years, *p* < 0.001, and had smaller burns but similar length of stay. Fifty percent of the 225 pavement group patients with full-thickness burns required skin grafts. There were 13 (6%) fatalities in the pavement group vs 1 (4%) in the MVC group, *p* = 0.01. Fatalities were secondary to sepsis, shock, cardiac, respiratory, or kidney complications. Compared to survivors, the non-survivors had a significantly higher % TBSA (10% vs 4%), *p =* 0.01, and lower Glasgow Coma Scores (10 vs 15), *p* = 0.002.

**Conclusion:**

There was a median 2 °F increase in ambient temperature since 1995. The increase in pavement burn admissions was multi-factorial: higher temperatures, population, and the number of older patients, with increased metropolis expansion, outreach, and urban heat indices. Pavement group was similar to the MVC group except for significantly older age and increased mortality. Morbidity associated with age contributed to increased mortality.

## Background

There are few literature articles describing burns sustained from body contact with naturally heated surfaces such as pavements, sidewalks, streets, and highways. Temperatures severe enough to contribute to burns from the environment have been reported, especially in heavily populated desert areas. The first accounts of pavement-street contact burns were the Arizona Burn Center case series of three motor vehicle crashes (MVCs) by Berens in 1970 [1], and the temperature and patient description in 1995 by Harrington et al. [2]. Additional contact pavement burn reports from this center were on pediatric feet in 2006 [3], and seizures in 2007 [4]. The aim of this study was to investigate the demographic characteristics of the recent significant increase in the number of pavement-street burns from 2016 to 2018 compared to those previously published in 1995 [2], which had reported 23 cases in a 6-year-period (1986-1992), and other studies in the literature. The hypothesis was that the increased number of pavement-street burns was due to higher ambient and naturally heated surface temperatures. In addition, we proposed that contact burns sustained in MVCs/road rash were significantly less severe than those which resulted from body contact with hot pavements-streets.

## Methods

### Sample population

This was a three-year retrospective burn center in-house registry study at the Arizona Burn Center. The study population consisted of patients who had contact burns as an initial admission mainly in the summer with late spring and early autumn months (May-September) of a three year (2016 to 2018) period. These burns were sorted into: pavement/street/highway/asphalt/bench: pavement group (n=225); status post MVC: MVC group (n=27); road rash: road rash group (n=15), and scald/hot object, etc.: other contact group (n=103). The database consisted of de-identified patients. This study was approved by the Maricopa Integrated Health System Institutional Review Board in Phoenix, Arizona.

### Previous temperature data

In 1947, studies by Moritz and Henriques [5–7] noted that second degree burns resulted when the contact surface temperature was 44^o^C (111^o^F) for 6 hours, 50^o^C (122^o^F) for 15 minutes, 60^o^C (140^o^F) for 5 seconds, and 65^o^C (149^o^F) for 2 seconds. In comparison, the American Burn Association [8] stated that hot tap water and scalds can cause third degree burns in 5 seconds at 60^o^C (140^o^F), and 1 second at 68^o^C (155^o^F). In Adelaide, Australia, Clifton et al. [9] documented similar natural surface temperatures for shaded and unshaded slate, metal, cement, sand, brick and bitumen. Rumney et al. [10] and Way [11] measured asphalt temperatures in different state locations in the 1970’s and 1980’s and found that the peak daily temperatures occurred around 3 pm. The hottest temperatures were in June and July with a mean daily asphalt temperature of 68^o^C (154.4^o^F) and a maximum daily asphalt temperature peak of 71.1^o^C (160^o^F) [10, 11]. In relation to climate change, it has been recorded that the state has warmed about 2^o^F in the last century [12]. Comparing the average annual number of days with maximum temperatures of 110^o^F or higher from 1981-2010 to 1896-2010, it was 19 days vs 11 days [13].

Harrington et al. [2] recorded their own temperatures on concrete, asphalt, steel, lawn, dirt and sand. They noted that ambient temperatures of 35^o^C to 37.8^o^C (95^o^F to 100^o^F) were necessary to produce a surface temperature of 44^o^C (111^o^F) to cause a cutaneous burn injury. In their study, asphalt and sand peaked at approximately 68^o^C (154^o^F), and dirt, cement and steel peaked at 58-60^o^C (136-140^o^F). Shade decreased the surface temperature on asphalt and cement peaks to 43^o^C (109^o^F) and 39^o^C (102^o^F) respectively [2]. Depending on the time, length of ground contact, and season, motorcyclists may have some protection from clothing (depending on what they are wearing).

### Months of May to September from 2016 to 2018 data collection

Daily ambient temperatures were obtained for the months of April through October for three years: 2016 to 2018 from the timeanddate.com website [14] with daily high and low temperatures at 6 am, 12 noon, and 6 pm. Data was also obtained to determine the highest ambient temperatures on the days when patients with pavement-street contact burns were admitted to the hospital compared to days when there were no admissions.

The authors utilized an IRT207 Heat Seeker™ Infrared Thermometer (2015 General Tools and Instruments, Secaucus, NJ; Montreal, Canada H9R 1E1) to measure the sidewalk, asphalt, and rock temperatures for a three-week-period (August 8 to August 31, 2018). This IRT was an 8:1 non-contact temperature measuring instrument using infrared technology and laser sighting. Features included a 4 digit backlit liquid crystal display (LCD), scan/hold function and auto power off (7 secs), a Laser Class 2 with an Output/Wavelength of <1mW @655nm, and repeatability of ± 1^o^C (± 1.8^o^F). The thermometer temperature readings were recorded in Centigrade and Fahrenheit with the thermometer held one inch off the surface according to manufacturer instructions. The emissivity of the instrument did not need to be set by the user.

### Statistical analysis

Statistical analysis was performed utilizing Statistica® (StatSoft, Tulsa, OK) descriptive statistics, one way- analysis of variance (ANOVA), with unequal N Tukey post-hoc comparisons, Maximum Likelihood Chi-squared tests. Comparisons were made between pavement, MVC, road rash, and other contact groups. The following demographic dependent parameters in the four independent groups were studied by one-way ANOVA statistics: age, percent of total body surface (%TBSA), procedures, length of stay (LOS) days, hospital charges, intensive care unit (ICU), injury severity score (ISS), Glasgow coma scale (GCS), body mass index (BMI), ventilator (vent) days, operating room (OR) visits, with ambient and natural surface temperatures by month and year. Maximum Likelihood chi-squared and Tukey was utilized for four study groups and pavement group subsets for: ethnicity, injury location, treatment, surgery, mortality, co-morbidities, payers, transport, graft use, ethanol/drug use, and cellulitis. In all cases, a *p* value < 0.05 was considered significant.

## Results

### Ambient and naturally heated surface temperatures

**Fig. 1:**
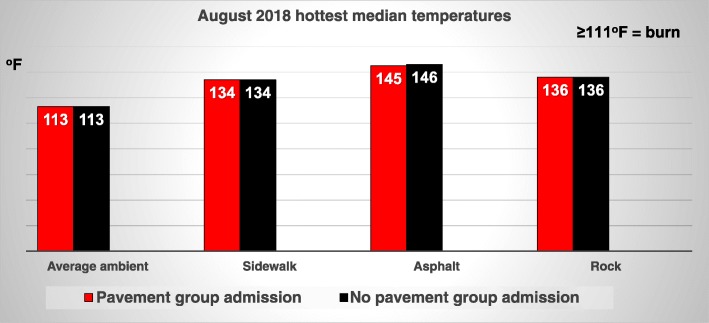
Streets of fire revisited 2016–2018: temperatures on pavement group admission days and non-admission days: August 2018 hottest median temperatures

The median ambient temperatures in the 1995 [2], 2006 [3], and present study periods were similar (Table 1). Figure 1 indicates that ambient temperatures were not higher on the days when the contact pavement group patients were injured and admitted to the burn center compared to days when there were no pavement group injury admissions. There was no statistical significance in the ambient temperature difference between the four groups or the three years in the current study.

**Table 1:** Median ambient and natural surface temperatures in degrees Fahrenheit and Centigrade: comparison of the literature with the present study

Ambient temperature comparison		Median (°F)	Range (°F)
1995 “Streets of Fire” [2]		106	100–113
1996 Mecca burnt feet on sand [15] 2000^a^ Friday mass in Saudi Arabia [16]		AV noon: 113	122–140
2006^b^ “Pediatric foot burns” [3]		–	86–106
Present study high noon (2016–2018)		106	84–119
Present study low noon (2016–2018)		100	73–115
High noon ambient median temperatures on the day of admission from May to September during 2016–2018			
Pavement group		108	86–119
Road rash group		100	100–110
MVC group		104	88–111
Other contact group		106	90–117
August 2018 (12 pm and 6 pm) (median) temperatures of pavement/asphalt/rock			
Pavement	12 pm, °F (°C)	129 (53.3)	98.0–140.0 (36.7–61.7)
	6 pm, °F (°C)	111 (43.1)	96.6–117.5 (35.9–47.3)
Asphalt	12 pm, °F (°C)	138.5 (59.5)	116.9–152 (41.8–68.7)
	6 pm, °F (°C)	124.5 (52.0)	111.3–132 (44.1–56.0)
Rock	12 pm, °F (°C)	124.1 (52.7)	98.6–138.7 (37.0–59.2)
	6 pm, °F (°C)	102.0 (37.6)	91.9–110.3 (32.7–43.6)

*AV* average, *MVC* motor vehicle crash

^a^Friday Muslim mass held at noon; slippers outside the mosque get misplaced

^b^Injuries occurred between 12 noon and 4 pm

**Fig. 2:**
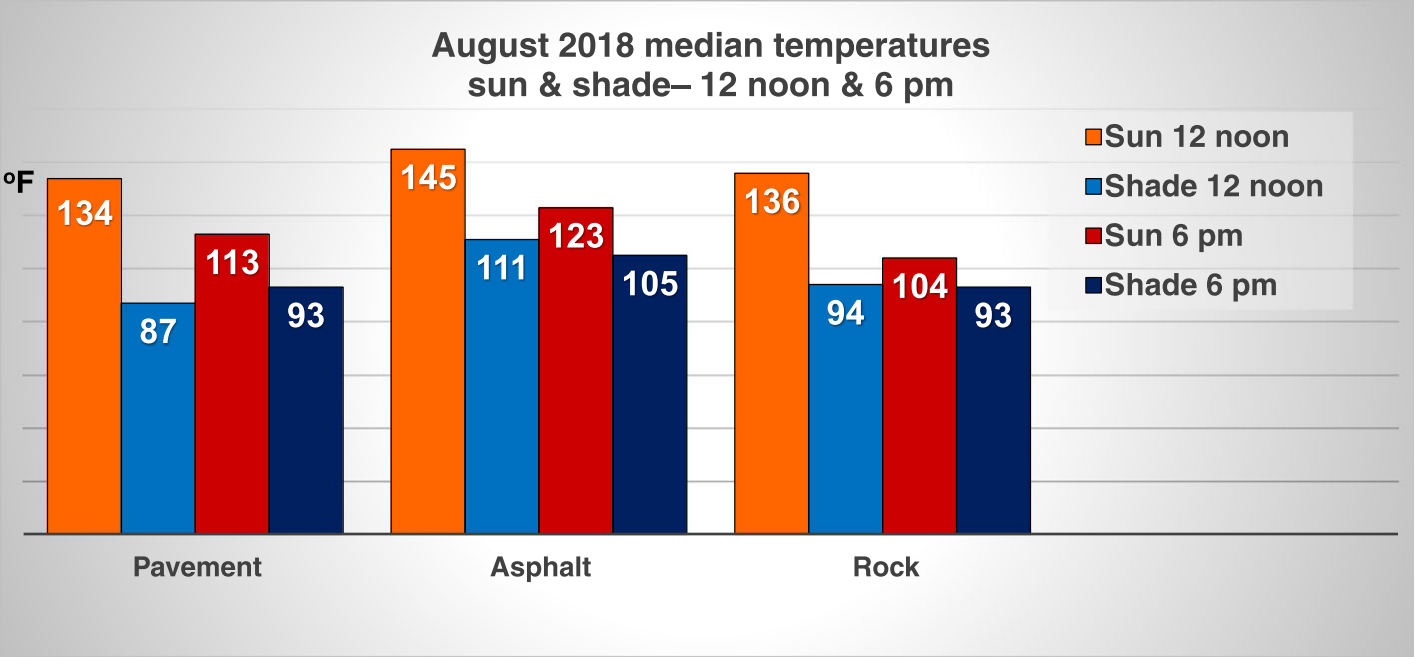
Streets of fire revisited 2016–2018: August 2018 median temperatures of pavement, asphalt, and rocks in the sun at 12 noon and shade at 6 pm

The pavement, asphalt, and rock surface temperatures were comparable to those recorded in the 1995 article, indicating that surface temperatures in the range of 95-100^o^F were sufficient to cause a cutaneous burn [2]. There were similar surface temperature results in August 2018 as seen in 1995, in reference to shade even during high noon temperatures, providing relief from the heat, except for the asphalt surfaces (Figure 2). Although the surface temperatures decreased by 6 pm, they were still hot enough to cause cutaneous contact burns.

### 2016–2018 population

**Fig. 3:**
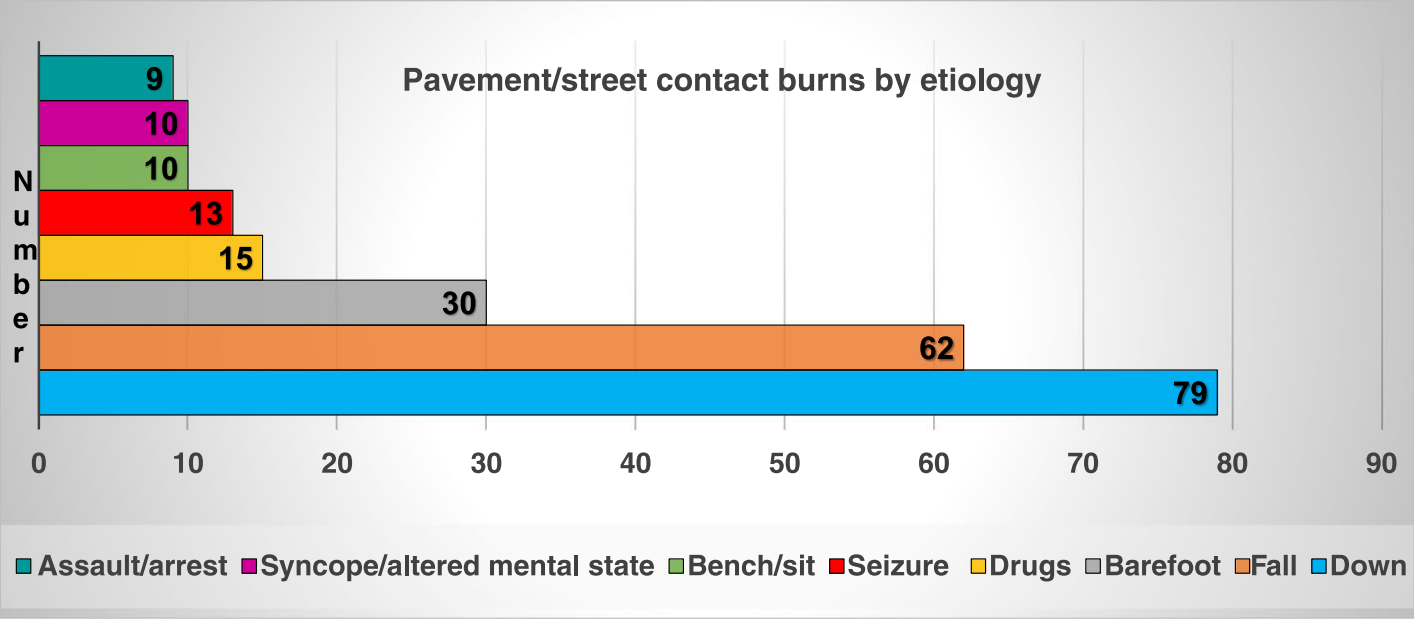
Streets of fire revisited 2016–2018: number of cases in each etiology subset of the pavement group

The majority of the patients in this study were those who were burnt by naturally heated surfaces such as pavement, streets, sidewalks, cement, highways, and rocks (Figure 3). The highest number of admissions (97) was in 2017. The demographic characteristics of the four study groups are shown in Table 2. Patients in the pavement group were significantly older than the patients in the MVC, road rash or other contact groups, *p =* 0.002. Compared to the other contact group, the pavement group had a significantly higher % TBSA (4% vs 1.5%), *p =* 0.004; longer LOS (12 days vs 4 days), *p* < 0.001; more procedures (5 vs 2), *p* < 0.001, and hospital charges (US$172,024 vs US$51,802), *p* = 0.005, with a significantly lower ISS score compared to the road rash group (1 vs 4), *p* <0.001. In the pavement group, 151 (67%) were Caucasian, 38 (17%) Hispanic, 23 (10%) African American, and 11 (5%) Native American; there was also one Hawaiian and one Asian.

**Table 2:** Demographic characteristics of contact burns (May to September, 2016–2018)

Parameters	Pavement	MVC	Road rash	Other contact	*P* value
Number	225	27	15	103	–
Age (years)	57 (1–97)	34 (15–65)	32 (6–59)	37 (0.1–92)	0.002
Sex (male/female)	155/70	18/9	11/4	64/39	0.6
% TBSA	4 (1–30)	7 (1–19)	8 (1–19)	1.5 (0.25–18)	0.001
Ethanol levels (mg/dl)	151 (24–395)	183 (37–364)	167 (54–280)	68 (40–236)	–
ISS	1 (1–34)	5 (1–43)	4 (1–5)	1 (0–10)	0.001
GCS	15 (3–15)	15 (3–15)	15 (7–15)	15 (1–15)	0.76
BMI, kg/m^2^	27 (16–66)	27 (18–102)	25 (17–53)	25 (10–47)	0.33
Hospital LOS (days)	12 (1–109)	12 (1–61)	11 (1–19)	4 (1–36)	0.001
LOS/% TBSA (days)	3	1.7	1.3	2.7	–
ICU LOS (days)	8 (1–69)	16 (1–50)	–	–	–
Ventilator (days)	8 (1–58)	16 (7–41)	–	–	–
Procedures (number)	5 (0–106)	10 (0–50)	11 (0–24)	2 (0–37)	0.001
Cellulitis, n (%)	22 (10%)	1 (4%)	0	10 (10%)	0.67
Hospital charges (US$)	172,024 (8128–5,468,380)	239,018 (7789–2,261,061)	190,616 (5717–305,053)	51,802 (3173–684,096)	0.005
Median hospital charges/hospital LOS (US$)	14,335	19,918	17,329	12,951	–
Median hospital charges/% TBSA (US$)	43,006	34,145	23,827	34,535	–
Mortality, n (%)	13 (6%)	1 (4%)	0	0	–
Ethnicity, n(%)					
Caucasian	151 (67%)	15 (56%)	9 (60%)	56 (55%)	–
Hispanic	38 (17%)	9 (33%)	3 (20%)	28 (27%)	–
African-American	23 (10%)	1 (4%)	0	4 (4%)	–
Native American Indian	11 (5%)	2 (7%)	2 (13%)	14(14%)	–
Hawaiian	1 (0.4%)	0	0	0	–
Asian	1 (0.4%)	0	1 (7.0)	0	–

Pavement group age was significantly increased compared to all groups. Pavement % TBSA, hospital LOS, procedures, and median hospital charges were significantly increased compared to the other contact group. Pavement ISS was significantly increased compared to the road rash group. There was no significant difference in hospital charges for the pavement vs the MVC group, *p* = 0.8. Data presented as median (range)

*% TBSA* percent of total body surface area, *LOS* length of stay, *ICU* intensive care unit, *ISS* Injury Severity Score, *GCS* Glasgow Coma Scale, *BMI* body mass index *MVC* motor vehicle crash

### Pavement group social characteristics

In the pavement group, 127 (56%) were single; 59 (26%) married; 13 (6%) divorced and 16 (7%) widowed. Of the 56+ years old patients in the pavement group, 61 (52%) lived alone and 43 (36%) lived with a spouse, partner or significant other compared to the age group 15-55.99 years, where 66 (67%) lived alone and 23 (22%) lived with a spouse, partner or significant other.

### Pavement group injury etiology

The different etiologies for the pavement burns are depicted in Figures 3. The five major reasons for injury are: **a** 79 “down” cases--(unknown reasons: could not get up, were unresponsive, etc.) median age 56 (range 15-94) years old, 4 (range 1-10.5) % TBSA; **b** 62 fall cases--median age 68 (range 44-97) years old, 4.5 (range 1-18) %TBSA; **c** 30 barefoot cases--median age 59 (range 1-79) years old, 1.5 (range 1-4) %TBSA; **d** 15 ethanol & drugs--median age 32.5 (range 24-60) years old, 4.3 (range 1-13) %TBSA; and **e** 13 seizures--median age 42 (range 19-66) years old, 4 (range 1-10.5) %TBSA. The highest number of pavement group patients were reported as “down” in the registry because the medical charts indicated that the reason for their being on the ground was unknown, or could not be determined by the paramedics who transported them to a medical facility. There was a statistically significant difference between the down vs barefoot patients in % TBSA (median 7 %TBSA vs 1.5 %TBSA), *p* <0.001, with increased procedures (median 8 vs 0), and operating room visits (median 2 vs 0). There was also a statistically significant difference in age for fall vs seizures (median 68 years vs 42 years), *p =* 0.006; fall vs down (median 68 years vs 56 years), *p =* 0.03; and fall vs ethanol & drugs (median 68 years vs 32 years), *p* < 0.001. Additional etiologies were for the following number of patients: drugs 15; ethanol 13; heat stroke 11, bike/scooter 11, parking lot 13, bus stop 7, wheelchair and mailboxes 6, outdoor city benches 3.

### Pavement group transport

The majority of the pavement group patients were transported by ground ambulance 136 (60%), public or private vehicle 75 (33%), and helicopter ambulance 13(6%). They arrived: directly from the scene 117 (52%); an emergency room 52 (23%); the burn center 30 (13%); and from another acute facility 25 (11%). Upon discharge, 103 (46%) were transferred to other facilities for rehabilitation or skilled nursing, 70 (31%) were discharged home, 17 (8%) were discharged home with home services, and 14 (6 %) had other dispositions.

### Pavement group injured body area

The majority of the areas affected in the pavement group were posterior trunk, buttocks, lower arms, legs and feet. The number of patients with involved areas were: 55 patients had posterior trunk with a median 3% TBSA; 52 had buttock injury with 1.5% TBSA; 63 patients had left lower arm involvement of 1% TBSA; 88 injured their legs with a median 1% TBSA, and 59 had feet injured with 0.5% TBSA. Compared to the other groups, multiple areas were affected. The majority of the pavement group had either: 71 of 225 (32%) patients - one area burnt; 51 (23%) - two areas; 50 (22%) - three areas, and 36 (16%) - four areas burnt.

### Pavement group surgery

One hundred thirteen of 225 (50%) pavement group patients required full thickness excision with skin grafting treatment vs 12 of 27 (44%) MVC group patients, *p* = 0.02. There were 6 (3%) patients in the pavement group who had full thickness burns and were treated without grafts compared to one (4%) in the MVC group. Treatment for non-extensive burns was given to 33 (15%) of the pavement group patients compared to the 2 (7%) of the MVC group.

### Pavement group comorbidities

The most common co-morbidities were medically treated hypertension, history of ethanol and/or drugs, psychiatric disorders, diabetes, current smoking, cardiovascular, kidney, liver, and respiratory disease (Table 3). Both the 15-55.99 and 56+ age patients were most frequently seen in the down and fall subsets. Common issues with drugs, seizures, smoking, and psychiatric illnesses were seen in the 15-55.99 age subset. The down category had the highest mortality rate of the pavement group: seven of 79 (9%); two had hypertension and five did not. The fall category had the second highest number of deaths: three of 62 (5%); one had hypertension and two did not. Older individuals who fell, stayed on the ground because they were not able to get up. The barefoot group had two fatalities (one with hypertension and another without hypertension).

**Table 3:** Number of comorbidities by history in pavement group 56+ years old patients (108) in the down, fall, and barefoot injury etiology

Comorbidity, n(%)	Down,41 (38)	Fall, 50 (46)	Barefoot, 17 (16)
Hypertension (treated), n(%)	18 (44)	29 (58)	10 (59)
Ethanol/drugs, n(%)	19 (46)	11 (22)	4 (24)
Diabetes, n(%)	7 (17)	10 (20)	13 (76)
Cardiac, n(%)	9 (22)	14 (28)	3 (18)
Psychiatric, n(%)	8 (20)	14 (28)	4 (24)
Respiratory, n(%)	11 (27)	9 (18)	5 (29)
Smoking, n(%)	8 (20)	10 (20)	3 (18)
Neurological, n(%)	5 (12)	7 (14)	2 (12)
Renal, n(%)	7 (17)	3 (6)	2 (12)
Dementia, n(%)	5 (12)	5 (10)	0

These are numbers for each comorbidity followed by the percentage in parentheses. Individual patients may be counted multiple times if they had more than one comorbidity

### Pavement group mortality

In 2016, the mortality in the pavement group was four of 58 (7%) patients; in 2017, four of 97 (4%); and in 2018, five of 70 (7%). There were no mortalities in the road rash or other contact groups except for one of eight (13%) in the MVC group in 2018. The 0-14.99 year age group had no mortalities. The 15-55.99 year age group had one mortality each in the pavement and MVC groups. Of the 120 patients in the 56+ age subset, 13 (11%) were deceased. There was a statistically significant increase in pavement group deaths compared to the road rash, MVC, and other contact groups, *p =* 0.01. In addition, the 56+ age subset had a significantly higher mortality compared to the 0-14.99, and 15-55.99 age subsets, *p* < 0.001.

The majority of fatalities in these patients resulted from sepsis, cardiogenic/septic shock, trauma, as well as major cardiac, respiratory, and kidney failure complications. The patients were injured from being down (8), falls (3), or barefoot (2). One patient had severe multiple trauma secondary to an MVC vs pedestrian incident. The GCS median was 10 (range 3-15). The median age was 74 (range 49-94) years old. Seven patients had acute kidney failure, 2 had end stage renal disease on dialysis, and one had chronic renal disease. Ten patients developed respiratory failure and several had either an aspiration or bacterial pneumonia or both. Nine patients developed either septic, hypovolemic, or cardiogenic shock. Six patients had medically treated hypertension and two had diabetes. The majority of the deaths were complications from co-morbidities patients arrived with, such as rhabdomyolysis, ischemic heart disease and congestive heart failure; five were withdrawn from treatment and received palliative care. While the burn injury precipitated the systemic inflammatory response, most fatalities could be attributed to the myriad of serious co-morbidities, which resulted in organ failure and complications.

Within the pavement group, 203 of 225 (90%) survivors had an ISS < 6 vs 9 with an ISS ≥ 6, *p* = 0.02. Ten of 13 (77%) non-survivors had an ISS of < 6 and three (23%) had an ISS of ≥ 6, *p* = 0.02. The demographic characteristics of the survivors and non-survivors in the pavement group are shown in Table 4. There was no statistically significant difference in age subsets, *p* = 0.2. There was a significantly increased %TBSA in the non-survivors vs the survivors, (10 %TBSA vs 4 %TBSA), *p =* 0.01, and a decreased GCS in the non-survivors vs the survivors (10 vs 15), *p =* 0.002.

**Table 4:** Pavement group survivor vs non-survivor comparison

Parameters	Pavement/asphalt/rock		*P* value
Status	Alive	Dead^a^	–
Number (%)	212 (94)	13 (6)	–
Age (years)	56 (1–97)	71 (35–94)	0.2
Sex (male/female)	148/64	6/7	–
% TBSA	4 (1–27)	10 (2–30)	0.01
Ethanol levels (mg/dl)	151 (24–395)	115 (24–205)	0.4
ISS	1 (1–34)	4 (1–16)	0.79
GCS	15 (3–15)	10 (3–15)	0.002
Body mass index (kg/m^2^)	27 (16–66)	31 (17–45)	0.99
Down time (min)	25 (1–150)	–	–
Hospital LOS (days)	12 (1–109)	17 (1–37)	0.99
ICU LOS (days)	5 (1–69)	8 (1–24)	0.7
Ventilator (days)	9 (1–58)	6 (1–24)	0.4
Procedure number	5 (0–106)	8 (0–38)	0.99
Hospital charges (US$)	152,598 (8128–5,468,380)	681,537 (49,242–1,490,924)	0.99
Median hospital charges/LOS (US$)	12,717	40,090	–
Ethnicity, n(%)			
Caucasian	139 (93)	10 (7)	–
Hispanic	36 (100)	0	–
African American	23 (100)	0	–
Native American Indian	9 (82)	2 (18)	–
Hawaiian	1 (100)	0	–
Asian	1 (100)	0	–

Data presented as median (range)

*% TBSA* percent of total body surface area, *LOS* length of stay, *ICU* intensive care unit, *ISS* Injury Severity Score, *GCS* Glasgow Coma Scale, *down time* time spent on the ground before rescue, *MVC* motor vehicle crash

^a^1 of 27 (4%) MVC cases was a non-survivor and is not included here

### Hospital charge payers

To determine which payers provided funding for hospitalization, we looked at the different subsets by age. Within the pavement group, there were few children aged 0-14.99. The major payers for the pavement group were medicare, medicaid, and private/commercial insurance. In the 15-55.99 subset, insurance payments were: medicaid for 65 of 102 (63.7%); medicare 14 (13.7%); commercial 14 (13.7%); self-pay 7 (6.9%); other 2 (2%). For the 56+ age group, insurance payments were: Medicare for 87 of 120 (73%); Medicaid 25 (21%); commercial 4 (3%); other 3 (3%). Table 2 shows the hospital charges by group and Table 5 shows the charges by age subsets.

**Table 5:** Demographic characteristics by age group

	Age group		
	0–14.99	15–55.99	56–100
Pavement/asphalt/rock			
Number (%)	3 (1 )	102 (45)	120 (54)
Age (years)	7 (1–9.5)	44 (15–55)	70 (56–97)
Sex (male/female)	2/1	76/26	77/43
% TBSA	1.5 (1–5)	4 (1–27)	4 (1–30)
Hospital LOS (days)	2 (1-12)	12 (1-109)	13 (1-62)
Hospital charges (US$)	44,600 (14,535–131,623)	14,192 (9195–5,468,380)	177,991 (8128–2,150,431)
Hospital charges/hospital LOS (US$)	22,300	1183	13,692
Hospital charges/% TBSA (US$)	29,733	3548	44,498
Mortality, n(%)	0	1 (1)	12 (10)
Road rash			
Number (%)	2 (13)	11 (73)	2 (13)
Age (years)	6 (6–7)	32 (21–47)	58 (56–59)
Sex (male/female)	1/1	8/3	2/0
% TBSA	6 (6–7)	10 (1–19)	4 (2–6)
LOS (days)	7 (2–11)	10 (1–19)	15 (12–17)
Hospital charges (US$)	105,355 (15,022–195,689)	190,616 (5717–305,053)	193,352 (192,277–194,426)
Hospital charges/LOS (US$)	15,051	19,062	12,890
Hospital charges/%TBSA (US$)	17,559	19,062	48,338
Mortality, n(%)	0	0	0
MVC			
Number (%)	1 (4)	21 (78)	5 (19)
Age (years)	14.6	27 (18–52)	58 (56–65)
Sex (male/female)	1/0	12/9	5/0
% TBSA	1	8 (1–19)	6 (1–9)
Hospital LOS (days)	3	12 (1–61)	12 (5–24)
Hospital charges (US$)	34,289	210,428 (7789–2,261,061)	244,684 (51,392–1,165,514)
Hospital charges/hospital LOS (US$)	11,430	17,536	20,390
Hospital charges/% TBSA (US$)	34,289	26,304	40,781
Mortality, n(%)	0	1 (4)	0

Data presented as median (range)

*% TBSA* percent of total body surface area, *LOS* length of stay, *ICU* intensive care unit, *ISS* injury severity score, *GCS* Glasgow Coma Scale, *MVC* motor vehicle crash

### Literature review comparison with the current study

A search through PubMed, Medline, Google Scholar, the Internet, and pertinent articles as references in appropriate studies was undertaken. Published English literature on pavement, highway, street, cement, gravel, sand, and naturally heated surfaces was compared with the current study data. Ten articles found on this topic were compared based on the number of cases, author, year, country, population, and burn size. Table 6 shows the demographic characteristics of patients in the literature. Religious cultural practices may contribute to foot contact burns as seen in Table 1 [15, 6]. Alcoholism were noted in four patients in the Harrington [2]: three assaults/arrests, and one heat stroke case. The "Streets of Fire" article about Phoenix in 1995 [2] and the Al-Qattan article on Saudi Arabia in 2000 [16] had more than 10 cases each and were statistically compared separately from the “other”, <10 cases reports [1, 15, 17, 18] group. The majority of cases were diabetic and/or barefoot pedestrians, although there were a few case reports of seizures, and syncope.

**Table 6:** Literature reports on pavement-street contact injury

Author	Date	Journal	Cases	Age (years)	Sex	Population	% TBSA
Berens [1], Phoenix, AZ, USA	1970	*Journal of the American Medical Association*	3	22	F	MVC vs pedestrian	10
				67	M	Fell out, moving car	
				82	F	MVC: thrown out	12
Vardy et al. [18], Beer Sheva, Israel	1989	*Burns*	1	79	F	Heatstroke	20
Harrington et al. [2], Phoenix, AZ, USA	1995	*Annals of Emergency Medicine*	23	3	M	MVC vs pedestrian	12
				3	M	Child abuse	3
				34	M	Assault	4
				33	M	Police restraint	10
				48	M	Police restraint	11
				0.75	M	Age extreme	1
				1.08	M	Age extreme	2
				22	M	Seizure	7
				34	M	Seizure	10
				35	M	Seizure	3
				63	M	Seizure	2
				46	M	Weakness	8
				31	M	Syncope	9
				29	M	Drugs	10
				46	F	MVC vs pedestrian	5
				40	F	Assault	2
				57	F	Heatstroke	4
				82	F	Heatstroke	7
				69	F	Fall, pavement	13
				30	F	Lumbosacral radiculitis	1
				48	F	Diabetic neuropathy	2
				64	F	Diabetic neuropathy	2
				78	F	Diabetic neuropathy	3
Fried et al. [15], Saudi Arabia	1996	*Burns*	1	58	M	Barefoot	1
Al-Qattan [16], Saudi Arabia	2000	*Burns*	12	1	M		NA
				2	M		
				52	M		
				41	M		
				50	M	Diabetes, PVD	
				55	M	Diabetes	
				46	M	Diabetes	
				48	M	Ddiabetes	
				52	M	Ddiabetes	
				53	M	Diabetes	
				61	M	Diabetes	
				41	M	Diabetes	
Sinha et al. [3], Phoenix, AZ, USA	2006	*Journal of Trauma*	34	NA	NA	Pediatric feet	≤ 1
Rimmer et al. [4] Phoenix, AZ, USA	2007	*Journal Burn Care & Research*	10	NA	NA	Seizures	
Silver et al. [17], Las Vegas, NV, USA	2014	*Journal of Wound Care*	7	71	M	Syncope	8
				37	M	Fall, bike	10
				77	F	Fall	7
				57	M	Passed out	10
				29	F	MVC vs pedestrian	20
				57	M	Homeless	7
				50	M	Homeless	10
Silver [27], Las Vegas, NV, USA	2015	*Journal Burn Care & Research*	72	NA	NA	Diabetes, altered mental status, toddlers	Average 6.7
Vega [23], Las Vegas, NV, USA	2019	*Journal Burn Care & Research*	173	NA	NA	Pavement burns	Average 7.7
Present study, Phoenix, AZ, USA	2019		225	Mean 56	155 male/70 female	Down, fall, barefoot, etc.	Mean 6

*AZ* Arizona, *NV* Nevada, *USA* United States of America, *MVC* motor vehicle crash, *PVD* peripheral vascular disease, *%TBSA* percent of total body surface area, *F* female, *M* male, *NA* not available

Four additional news media articles on the World Wide Web depicted an adult and three children with bare feet who developed plantar foot burns: a) Citrus Heights, CA, 28 years old with approximately a 20% TBSA pavement burn sustained during a police arrest [19]; b) Huntington Beach, CA, 16 month old playing on the pavement outside the home [20]; c) Ipswich Park, Australia, 18 month old burned by a metal ground plate covering a service pit in a park [21]; d) Perth, Australia, 14 month old playing on the pavement outside the home [22].

**Fig. 4:**
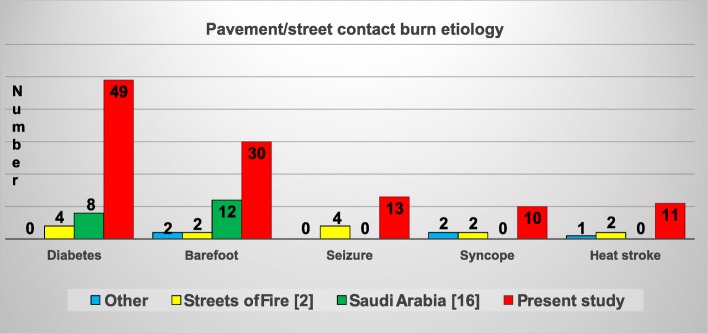
Literature reports 1970–2018: comparison of the current burn center cases with other case reports [1, 15, 17, 18], Streets of Fire [2], and Saudi Arabia [16] by pavement-street burn etiology

**Fig. 5:**
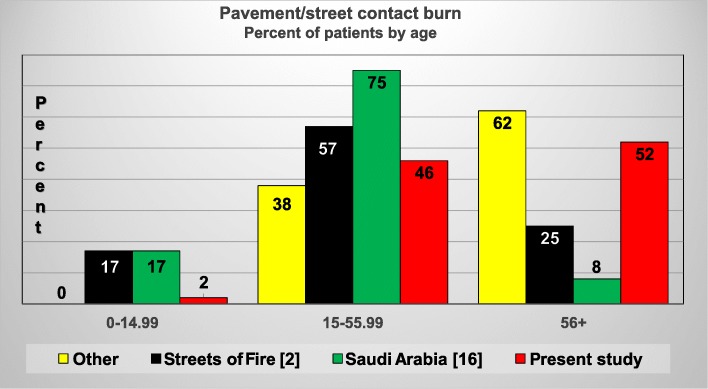
Pavement-street contact burn literature reports 1970–2018: age comparison of the current burn center cases with other case reports [1, 15, 17, 18], Streets of Fire [2], and Saudi Arabia [16]

There were very few children in the present pavement group and in the literature reports, especially since 2006. Compared with previous published series**,** the present study had a similar number of assault/arrest, syncope, seizure and heat stroke cases. However, the number of diabetic and barefoot/pedestrian cases at the burn center were significantly increased compared to the literature (Figure 4). Al-Qattan (Saudi Arabia) [16] reported the highest percentage of injuries in the 15-55.99 years old age subset. The present study and the “other” , single case reports, [1, 15, 17, 18] had the highest percent of 56+ years old patients compared to the “Streets of Fire” [2] and Saudi Arabia [16] cases (Figure 5).

## Discussion

The results of the present study compared to the 1995 Harrington et al. “Streets of Fire” [2] report indicate that the increase in pavement-street contact burns from 2016 to 2018 was multi-factorial: higher temperatures, population, number of seniors, with increased metropolis expansion, outreach, and urban heat indices for Phoenix. The median 2^o^F increase in ambient temperature between the “Streets of Fire” [2] article and the current study may be reflective of the climate change records, which indicate that the state has warmed up by 2^o^F in the last century [12]. In the 1995 study [2], median ambient temperatures were 106^o^F (range 100-113^o^F) compared to 108^o^F (range 86-119^o^F) highest noon temperature in the current study. In Phoenix, the maximum daily ambient temperature on the day of injury admissions was not increased compared to days when there were no pavement-street burn admissions. This center experienced more of these admissions in the target months of summer, with late spring and early autumn (May to September) during almost constant daily high temperatures above 95-100^o^F compared with January to April and October to December during the three year period. The present study does not reflect the most recent pavement burn report of exponential increase in burn pavement admissions with increasing temperatures seen in Las Vegas, Nevada [23]. Contact burns sustained in motor vehicle crashes were significantly less severe than those from body contact with hot pavements-streets as evidenced in the statistically significant increased mortality in the pavement group compared to the MVC group (6% vs 4% respectively). However, the pavement and MVC groups were similar in median levels of LOS, GCS, BMI, and ethanol use.

From a population of 1.135 million in 1995, the current 2017 estimate is 1,626 million, which makes Phoenix the 5^th^ largest city in the United States, replacing Philadelphia, PA [24]. As of 2018, the major Phoenix ethnicity was Caucasian (44%), followed by the Hispanic contingent of 41%, African American 7%, Asian 4%, and other 4% [25]. In comparison, the pavement group had only 17% Hispanic patients with burn injuries. The present study did not determine why there was such a difference between the Hispanic population and the Hispanic pavement-street contact injuries. One possible reason is that the burn center serves a Phoenix metropolitan population of more than 4 million individuals, Arizona and neighboring states, not all of which have a high Hispanic population. Since 1995, as the population and technological innovations increased, there was an expansion of burn center services, advertisements, public awareness through research with community education, and extended outreach to the adjacent state communities. The population growth may also have contributed to the increase in pavement-street contact burns.

Two Phoenix populations have been previously reported as pavement burn injuries: pediatric patients with foot burns and seizure patients. The 2006 study by Sinha et al. [3] reported that 34 of 182 children had burnt feet treated between May 2000 and August 2005. Of these, only one required hospitalization and grafting; all the others were treated as outpatients [3]. In August 2005, the Arizona State Department of Health Services instituted mandatory sun safety education in schools and required that the *SunWise* program sponsored by the United States Environmental Protection Agency be taught in all public elementary schools [26]. This prevention program may have been effective in the pediatric population since we saw so few children in this study.

In 2007, Rimmer et al. [4] reported a similar number of seizure patients to that seen in the current study group. In a period of 5 years, there were 32 patients with seizures admitted of whom 10 were burnt falling on hot pavements [4]. The report noted that the seizure patients had a mean±standard deviation (SD) of 3.8±2 %TBSA compared to this study group of seizure patients who had a mean±SD of 4.6±3 %TBSA; their average length of stay was 6.8 days compared to the median of 14 days for the present study seizure patients. There was no age stated for the reported seizure patients for comparison.

An additional locale where there have been pavement-street burns has been Las Vegas, Nevada. The findings in their seven patients have been included with the other group articles in the literature review (Table 6) [15]. Hospital charges are an important consideration and were assessed in a Las Vegas study comparing patients with pavement burns with/without altered mental status (AMS) from May 2008 to September 2012 [27]. The patients had a median 6.7 %TBSA size burn with a median hospital charge of US$ 13,276/%TBSA and a median LOS of 12 days [27]. In comparison, although the current study pavement group had a median of 4% TBSA, an LOS of 12 days, and a median hospital charge for the hospitalization of US$172,024, the hospital charges/%TBSA were US$43,006 and hospital charges/LOS were US$14,335. The different charges between the two institutions may be a result of multiple factors that were not known for this comparison, such as age, co-morbidities, complications, the frequency of third degree burns, etc. Recently, Vega et al [23] reported on 173 pavement-related burn injuries (2013-2017). More than 88 % of these injuries occurred when the ambient temperature was ≥ 95^o^F [23]. They also noted that as the ambient temperature increased, pavement burn injury admission increased exponentially [23].

Currently, there is much interest in assessing why some cities are hotter than others. One assessment method is the use of the urban heat island (UHI) index for determining how hot a city might be to live in. Debbage and Shepherd [28] from the University of Georgia investigated the 50 most densely populated cities in the United States and determined that the UHI intensity in Phoenix was 0.52℃ to 0.91 ^o^C compared to Las Vegas, which had a lower UHI intensity of -1.37℃ to - 0.44^o^C. One of the reasons that Las Vegas has a lower UHI intensity is that the city consists of approximately 7.5% barren land compared to Phoenix, which is more densely populated with buildings and thoroughfares and has less than 1% empty land [28]. In addition, Las Vegas is positioned at a higher above sea elevation (2,011 feet) [29] compared to Phoenix at (1, 200 feet.) [30]. The higher the elevation, the cooler the ambient temperature gradient.

Climatologists and city engineers found that adjacent densely populated and sprawling city development both contribute to the UHI intensity, and that non-contiguous urban development separated by green spaces may lower the UHI intensity [28]. Another study recommended that city planners include more vegetation adjoining new construction and development to decrease the UHI intensity [31]. Since the “Streets of Fire” in 1995 [2], Phoenix has expanded in size with an increased population density, and increased concrete, asphalt and other surfaces absorbing and retaining radiant energy during the day, before the cooling off in the evening, when the surface heat is dissipated into the atmosphere. Another variable which contributes to population density and expansion of resource needs is the human element.

There were 225 pavement group cases, the majority of which were due to being “down” (time spent on the ground before getting rescued), falls (not being able to get up), and bare feet. Compared to the other groups, the pavement group was: older, 120 (53%) patients were 56+ years old, *p* <0.0002, and had smaller %TBSA but similar lengths of stay. There were 13 (6.0%) fatalities in the pavement group vs one (4.0%) in the MVC group. Co-morbidities were a major contribution to the mortalities in the pavement group elder population. They were the largest component of the down and fall subset. In this contingent, it was frequently unknown how long the patients were exposed to the hot pavement, how many were dehydrated, or suffered heat stroke, seizures, or syncope at the time of their injury. Of the fatalities, the major co-morbidities in the present study were acute renal and pulmonary failure and heart disease. The combination of hypertension treated with diuretics during the hot summer months may have contributed to the renal disease and mortality. A recent article indicated a negative impact of high ambient temperatures on kidney function in patient >75 years old medicated on common hypertensive drugs [32].

Nationally, there were approximately 35 million individuals 65+ years old in the United States in 2000, and that number increased to 46.2 million in 2014 because of the “baby boomer” generation [33]. It is projected to increase to 98.2 million in 2060 [33]. In 2015, Maricopa County had an estimated four million residents, of whom nearly 25% were 55+ years old [33].The biggest contingent was that of the 60+ years old seniors at 767,477 individuals [33]. In this study, the patients most at risk for falls and pavement burns were patients 56+ years old.

It has to be noted that these aged individuals will continue to settle and populate this area because of the climate, thereby escalating the prospect of continued increases in pavement-street contact burns. Utilizing the wisdom from the past, a *SunWise* program for seniors through the State Department of Human Services can be developed and promoted on TV, libraries, shopping malls, and senior centers for public health education to reduce the “down, fall, and barefoot “ pavement-street contact burn injuries.

## Limitations

This was a small three-year study of the burn center registry information which is provided to the National Burn Repository of the American Burn Association. As a retrospective review, bias may have been introduced due to the medical information obtained from hospital records and coder interpretation of the medical charts. The temperature readings of the heated surface areas were only three weeks in August; the hottest surface temperatures in June and July were not available for comparison. There were also few MVC and road rash cases for comparison

## Conclusion

Pavement burns have increased since the 1995 publication of the “Streets of Fire”. The majority were due to “down time spent on the ground before getting rescued,” falls, and barefoot excursions. The ambient and surface temperatures have not increased significantly since 1995, but reflected the median 2^o^F increase in ambient temperature that has contributed to state warming in this century. The majority of patients in these groups have been older adults, 56+ years old. With a higher death rate among the patients, pavement burns were significantly more severe than MVC contact burns. Because of baby boomers, it is projected that the senior population will be increasing in the future. Cities with high potential for pavement-street contact burns in the summer need to be prepared for the attendant health services and education this population will require. In addition, future city and desert area expansion may benefit from proliferation of green spaces throughout the city and outlying areas to decrease the UHI effect. Future comparison studies of ambient and surface temperatures in select city developments with/without green spaces may be warranted.

## Data Availability

Data and materials are available from the corresponding author.

## Abbreviations

AA: African American
ANOVA: Analysis of variance
BMI: Body mass index
F: Female
GCS: Glasgow Coma Scale
ICU: Intensive care unit
IRB: Institutional Review Board
ISS: Injury Severity Score
LCD: Liquid crystal display
LOS: Length of stay
M: Male
MVC: Motor vehicle crash
OR: Operating room
%TBSA: Percent of Total body surface area
UHI: Urban Heat Island Index
Vent: Ventilator

## References

[1] Berens James J. (1970). Thermal Contact Burns From Streets and Highways. JAMA: The Journal of the American Medical Association.

[2] Harrington William Z, Strohschein Bonnie L, Reedy David, Harrington § Jennifer E, Schiller William R (1995). Pavement Temperature and Burns: Streets of Fire. Annals of Emergency Medicine.

[3] Sinha Madhumita, Salness Rebecca, Foster Kevin N., Fenn Robin, Hannasch Christa (2006). Accidental Foot Burns in Children From Contact With Naturally Heated Surfaces During Summer Months: Experience From a Regional Burn Center. The Journal of Trauma: Injury, Infection, and Critical Care.

[4] Rimmer Ruth B., Bay R Curtis R., Foster Kevin N., Jones Melanie A., Wadsworth Michelle, Lessard Collette, Mathieson Kathleen, Caruso Daniel M. (2007). Thermal Injury in Patients With Seizure Disorders: An Opportunity for Prevention. Journal of Burn Care & Research.

[5] Henriques FC, Moritz AR. Studies of thermal injury. I. The conduction of heat to and through skin and the temperatures attained therein: a theoretical and experimental investigation. Am J Pathol. 1947;23:530–549. PMC1934298 19970945

[6] Moritz AR, Henriques FC. Studies of thermal injury. II. The relative importance of time and surface temperature in the causation of cutaneous burns. Am J Pathol. 1947;23:695–720. PMC1934304 19970955

[7] Moritz AR. Studies of thermal injury. III. The pathology and pathogenesis of cutaneous burns: an experimental study. Am J Pathol. 1947;23:915–941. PMC1934331 19970971

[8] Burn Foundation: http://www.burnfoundation.org and water temperatures. Accessed 26 Aug 2019.

[9] Clifton Thomas, Khoo Teng-Wei, Andrawos Alice, Thomson Sumana, Greenwood John Edward (2016). Variation of surface temperatures of different ground materials on hot days: Burn risk for the neuropathic foot. Burns.

[10] Rumney TN, Jimenez RA (1969). Pavement temperatures in the Southwest Highway. Research Record.

[11] Way GB. Environmental factor determination from in-place temperature and moisture measurements under Phoenix pavements. Arizona Department of Transportation, 1980; Report #FHWA/AZ-80/157.

[12] EPA What climate change means for Arizona. August 2016; EPA 430-F-16-005. https://19january2017snapshot.epa.gov/sites/production/.../2016.../climate-change-az.... Accessed 19 Jan 2019.

[13] Facts about 100 degree temperatures for Phoenix. http://southwestweather.com/wx/wx100degreeday.php. Accessed 19 Jan 2019.

[14] https://www.timeanddate.com/weather/usa/Phoenix/historic?month=6&year=2016 (year = 2017 and year = 2018) Accessed 1 April- September 30 2016 to 2018.

[15] Fried M., Kahanovitz S., Dagan (Reiss) R. (1996). Full-thickness foot burn of a pilgrim to Mecca. Burns.

[16] Al-Qattan M.M. (2000). The “Friday Mass” burns of the feet in Saudi Arabia. Burns.

[17] Silver A.G., Zamboni W.A., Baynosa R.C. (2014). Operative management of acute pavement burns: a case series. Journal of Wound Care.

[18] Vardy D.A., Khoury M., Ben-Meir P., Ben-Yakar Y., Shoenfeld Y. (1989). Full skin thickness burns caused by contact with the pavement in a heat-stroke victim. Burns.

[19] Hubert C. He suffered severe burns at the hands of police. Now he wants more than $26 million. (Citrus Heights, CA) Sacramento Bee, February 07, 2018 04:00 AM. https://www.sacbee.com/news/local/article198742299.html. Accessed 2 May 2019.

[20] Ludwig A. Hot Pavement Burns Feet; 2nd Degree Burns In Seconds, Southland Mom Says. PATCH; Jun 23, 2016 | Updated Aug 28, 2017. https://patch.com/california/newportbeach/huntington-beach-oc-mom-warns-dangers-2nd-degree-burns-hot-pavement. Accessed 2 May 2019.

[21] Riga R. Toddler suffers second degree burns from metal plate in Ipswich Park, ABC NEWS. November 8, 2018. https://www.abc.net.au/news/2018-11-08/toddler-burns-feet-standing-on-metal-plate-ipswich-park/10477340. Accessed 2 May 2019.

[22] Scanlan R. A Perth mum has warned about the dangers of hot pavement after her 14-month-old suffered severe burns to both his feet. news.com.au . January 17, 2019. https://www.news.com.au/lifestyle/parenting/kids/toddler-suffers-awful-burns-to-feet-after-walking-on-hot-path/news-story/4e9ff68714a44cbdd72cd5683e1dccd3. Accessed 2 May 2019.

[23] Vega Jorge, Chestovich Paul, Saquib Syed, Fraser Douglas (2019). A 5-Year Review of Pavement Burns From a Desert Burn Center. Journal of Burn Care & Research.

[24] Phoenix, Arizona Population History 1950 - 2017. https://www.biggestuscities.com/city/phoenix-arizona. Accessed 26 Aug 2019.

[25] Race and Ethnicity in Phoenix, Arizona. Updated on September 4, 2018, v1.1.d365c65b1203feeabd268194a484a408c4d69da0. https://statisticalatlas.com/place/Arizona/Phoenix/Race-and-Ethnicity Accessed 13 Sept 2019.

[26] SunWise Program. http://www.azdhs.gov/phs/sunwise. Accessed 9 January 2019.

[27] Silver Andrew G., Dunford Gerrit M., Zamboni William A., Baynosa Richard C. (2015). Acute Pavement Burns. Journal of Burn Care & Research.

[28] Debbage Neil, Shepherd J. Marshall (2015). The urban heat island effect and city contiguity. Computers, Environment and Urban Systems.

[29] Geographic coordinates of Las Vegas, NV, USA. https://dateandtime.info/citycoordinates.php?id=5506956. Accessed 12 Sept 2019.

[30] Geographic coordinates of Phoenix, AZ, USA. http://dateandtime.info/citycoordinates.php?id=5308655. Accessed 6 May 2019.

[31] Wang Chuyuan, Myint Soe, Wang Zhihua, Song Jiyun (2016). Spatio-Temporal Modeling of the Urban Heat Island in the Phoenix Metropolitan Area: Land Use Change Implications. Remote Sensing.

[32] Sagy Iftach, Vodonos Alina, Novack Victor, Rogachev Boris, Haviv Yosef S., Barski Leonid (2016). The Combined Effect of High Ambient Temperature and Antihypertensive Treatment on Renal Function in Hospitalized Elderly Patients. PLOS ONE.

[33] Wolfersteig W, Funke MDL, Yoder G, Kasunic ML. Older adult needs in Maricopa County. ASU- Southwest Interdisciplinary Research Center. 2017;1-57.

